# User involvement in regulation: A qualitative study of service user involvement in Care Quality Commission inspections of health and social care providers in England

**DOI:** 10.1111/hex.12849

**Published:** 2018-12-07

**Authors:** Emma Richardson, Kieran Walshe, Alan Boyd, Jill Roberts, Lillie Wenzel, Ruth Robertson, Rachael Smithson

**Affiliations:** ^1^ Alliance Manchester Business School University of Manchester Manchester UK; ^2^ The King's Fund London UK; ^3^ Present address: University of Leicester Leicester UK; ^4^ Present address: National Children's Bureau London UK; ^5^ Present address: Gold Coast Health Queensland Australia

**Keywords:** Care Quality Commission, health care, inspection, regulation, social care, user involvement

## Abstract

**Background:**

High profile failures of care in the NHS have raised concerns about regulatory systems for health‐care professionals and organizations. In response, the Care Quality Commission (CQC), the regulator of health and social care in England overhauled its regulatory regime. It moved to inspections which made much greater use of expert knowledge, data and views from a range of stakeholders, including service users.

**Objective:**

We explore the role of service users and citizens in health and social care regulation, including how CQC involved people in inspecting and rating health and social care providers.

**Design:**

We analyse CQC reports and documents, and 61 interviews with CQC staff and representatives of groups of service users and citizens and voluntary sector organizations to explore the place of service user voice in regulatory processes.

**Results:**

Care Quality Commission invited comments and facilitated the sharing of existing service user experiences and engaged with representatives of groups of service users and voluntary sector organizations. CQC involved service users in their inspections as “experts by experience.” Information from service users informed both the inspection regime and individual inspections, but CQC was less focused on giving feedback to service users who contributed to these activities.

**Discussion and conclusions:**

Service users can make an important contribution to regulation by sharing their experiences and having their voices heard, but their involvement was somewhat transactional, and largely on terms set by CQC. There may be scope for CQC to build more enduring relationships with service user groups and to engage them more effectively in the regulatory regime.

## 1 INTRODUCTION

In recent years, there have been a series of high profile failures of care in the NHS in England, and subsequent public inquiries have raised serious concerns about how well systems to oversee, regulate and hold to account health‐care professionals and organizations have worked.^1^, ^2^, ^3^ In response to recommendations from the Francis inquiry report,^2^ the Department of Health announced policy changes intended to ensure that poor care would be detected and acted upon.^4^ The Care Quality Commission (CQC) overhauled the way it regulated and inspected health and social care providers,^5^ moving to inspections which made much greater use of expert knowledge, data and views from a range of stakeholders, including service users. Performance was rated using a four‐point scale (outstanding, good, requires improvement or inadequate) and detailed narrative reports about providers were published following each inspection.^6^

During these reforms, public consultations^5^, ^7^ revealed shortcomings in CQC's public engagement strategy. In 2013, CQC sets an ambition to build better relationships with the public, to “promote greater public understanding and awareness of our work, improve our public information, improve how we listen to and act on people's views and experiences of care, and involve more people in our work”^6^ p. 14. Furthermore, it said it would also inspect how service users, citizens and their representatives were engaged, and involved in improving services.^6^ CQC does include service users or lay people as members of inspection teams, commonly termed “experts by experience.” As carers, or previous or current users of services, experts by experience are considered better positioned to elicit experiences from those using the service under review.^8^

The Francis inquiry also found that bodies responsible for patient, public and local scrutiny had been preoccupied with constitutional and procedural matters and consequently had failed to represent service user interests.^4^ Government had already legislated in 2012 to establish Healthwatch as a national body and a network of local authority‐commissioned services to listen to and share people's views of health and social care^9^ and it undertook to ensure both national and local Healthwatch were centrally engaged in CQC's inspection and rating process.^4^

Research has shown the importance of and potential for service user and citizen voice in regulatory activities,^10^, ^11^, ^12^ where voice refers both to people commenting on care received and being involved in the planning and provision of services and regulation, through local and community networks.^13^ Individual service user complaints are valuable to regulators and have previously highlighted failures in care, even if not always acted upon.^10^ Involving service users can improve institutional reviews of providers and services, by bringing legitimacy and accountability to the decision‐making process.^10^, ^12^ Regulators have been advised to capitalize on existing involvement activities and networks and to ensure any additional activities are tailored to regulatory goals.^10^

However, the arrangements for service user voice in health‐care regulation are not without criticism. Some question whether regulators really value patients as a source of information^10^ and others argue that the quality of the information gathered during institutional review from service users and citizens is very dependent upon the skills of the inspection team.^14^ The use of the term “expert by experience” has been challenged, as using a service or caring for someone might not necessarily qualify someone as an expert or as a lay assessor.^8^, ^15^ Additionally, professional hierarchies in inspection teams can make the integration of lay members difficult, affecting how well their voice is heard.^16^ It has been suggested that service user involvement in inspection may largely serve to add credibility to inspection judgements rather than genuinely promote service user experience within the inspection process.^8^

These developments should be set in the context of a substantial wider literature on service user voice in health and social care, which conceptualizes voice as both individually and collectively organized and heard.^17^ Individual voice comes from service users being involved with or interacting with health‐care professionals, as clients of health‐care organizations and as citizens who are entitled to access NHS health‐care services. Collective voice comes from groups of service users, care givers and citizens who, as lay stakeholders, provide representation on broader health issues faced by the segments of the population they represent. Individual voice, through lodging a complaint for example, can have a large impact at the micro level, improving care for those individuals but not necessarily leading to system level or policy changes. However, collective voice can lead to change at the system level which may have a wider and more enduring impact, for more people.^18^

**Figure 1:**
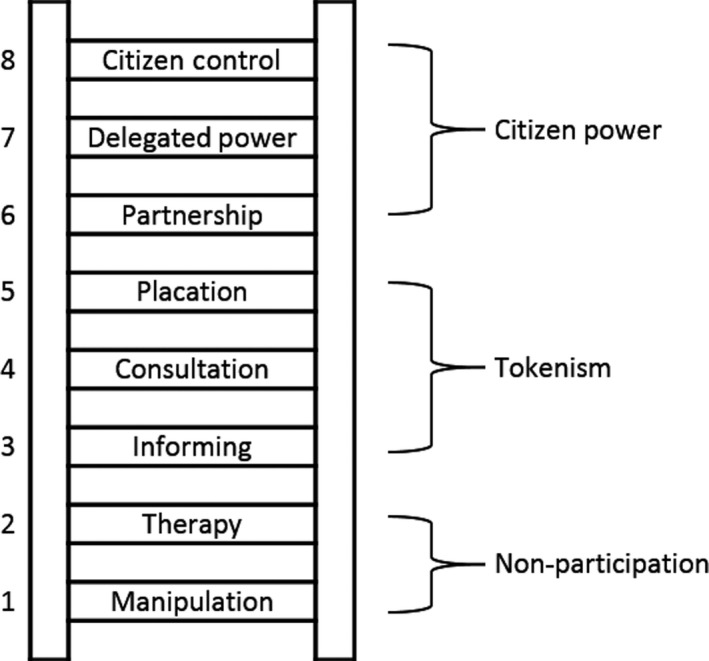
Arnstein's ladder of participation^19^

Arnstein's ladder of citizen participation^19^ has been widely used by researchers, policy makers and practitioners in conceptualising user participation. Figure 1 depicts a number of levels of citizen participation, in three main categories—from “non‐participation” through “tokenism” to “citizen power.” Non‐participation involves those with power attempting to educate or manipulate users but not really to involve them at all. What Arnstein describes as tokenism comprises efforts to inform users and consult them, but on terms framed or set by those in power, and in so doing to placate or reassure them. Citizens begin to have some influence, but those with power still have the final say, maintaining the status quo. At the top of the ladder, collective voice becomes “citizen power.” Citizens’ negotiate with the power holders, share responsibility for decision making and occupy key decision‐making positions. For almost 50 years, this framework has been widely used to understand patient and public involvement in the planning and provision of health care.^20^, ^21^ Yet, it has received criticism for being implicitly normative, suggesting progression upward towards genuine participation is desirable.^22^ Instead of focussing on the shift in power from one party to another, there is value in considering the impact service user involvement has at various levels as part of a wider system of participation.^23^

The aim of this paper is to present an inductive analysis of the role of service users and citizens in health and social care regulation, the first such investigation of this topic. We use Arnstein's ladder of participation to frame our understanding of CQC's involvement of people in the inspection and rating of health and social care providers.

## 2 METHODOLOGY

This paper draws on data collected between 2016 and 2017 as part of an evaluation of the effects of the CQC's new inspection and rating system on provider performance in England. Specifically, we focus on the role of service user voice in the inspection and rating of health and social care providers.

Our qualitative fieldwork focused on four care sectors: acute care, mental health, adult social care and general practice. Members of the research team observed comprehensive inspections in each of these sectors to gain an understanding of the research context.^24^ We also analysed selected CQC publications, policies, guidance and internal documents to understand whether and how user voice was incorporated into CQC's regulatory processes. The fieldwork took place in six geographic case study areas, loosely based on Clinical Commissioning Group (CCG) boundaries, and chosen to represent a variety of rural and urban contexts. In each area, at the time of our fieldwork there was a mix of organizations that had been inspected, with varying resulting ratings, and some which had not yet been inspected.

Interviewees were purposefully sampled according to their organization and job role.^25^ Interviews were conducted either face to face or by telephone depending on interviewee availability and preference. We interviewed a total of 61 interviewees, including 52 people from the six case study areas (see Table 1). The interviewees comprised 32 CQC staff from across the care sectors, seven representatives from local Healthwatch and 22 representatives of service user groups and voluntary organizations. For this last group, we sampled individuals who chaired or participated in Patient Participation Group (PPG) meetings and local branch officers of national charities which represent the patient voice. These people should have a more widely informed view of user involvement than individual service users, members of the public or lay inspection staff (experts by experience) would have in an individual capacity.

**Table 1:** Interviewees by case study area

	CQC inspection staff	PPG chairs and charity officers	Healthwatch representatives	Total
Area A	3	5	1	9
Area B	4	3	1	8
Area C	5	5	1	11
Area D	4	2	2	8
Area E	4	3	1	8
Area F	4	3	1	8
Total	24	21	7	52^a^

a We also interviewed eight CQC policy staff who were not area based, and one national representative of a PPG, making a total of 61 interviewees.

For CQC staff, questions focused on how the process of inspection and rating was intended to drive improvements in the quality of care of provider services, including questions concerning the place and use of service user voice. We also probed for reflections on how this was working in practice, including any unintended impacts. For Healthwatch and service user group interviewees, questions focused on how information CQC published was used, if service users and groups were involved with CQC or local provider organizations as they prepared for inspection, as they were being inspected or after the inspection outcome. We also asked about the nature and quality of the relationships between service user groups and CQC, and how inspection and rating impacted on service user experiences.

Informed consent was obtained for all interviews. The interviews were recorded, transcribed and coded by the research team in Dedoose, a qualitative software tool.^26^ Two members of the research team coded the interviews using an inductive, data driven approach^27^ allowing emerging themes to be identified. During analysis, ongoing discussion of themes and interpretation within the research team ensured analysis and interpretation was grounded in the interview data.^28^

## 3 FINDINGS

In this section, we present the main themes which emerged from our document analysis and interviews with CQC staff and service user group representatives. Arnstein's ladder of participation contains three main levels: the rungs at the lower end are categorized as “non‐participation,” those in the middle are labelled “tokenism,” and those at the higher end are designated “citizen power.” We find that the involvement of people in CQC's inspection and rating regime falls largely within the middle rungs of the ladder, informing, consultation and placation. We would note that the terms lower, middle and higher are used to relate CQC's service user involvement activities to Arnstein's ladder, but we do not presume that higher levels are necessarily preferable.

First, we examine how CQC draws on existing sources of service user voice, by gathering available data from various stakeholders prior to an inspection. Second, we explore how CQC engages with individual service users around the time of an inspection, to gather data relevant to the areas to be inspected. Third, we describe how CQC involves some service users directly in the inspection process, through its “experts by experience” programme. Fourth, we examine how CQC engages with service user groups to consult and gather collective voice, often less directly linked to a specific inspection. Finally, we explore how CQC provides information and feedback to service users after inspection, and the views of service users on the outcomes of inspections.

### 3.1 Gathering existing service user voice

From our review of documents and interviews with CQC staff, we found that CQC invited general information, such as compliments and complaints, from various stakeholders prior to inspection of a service or provider. Participation at this lower‐middle level included asking individual users to contact them; leaving comment cards in prominent locations; asking local and national partners such as Healthwatch to share any information they have received; and requesting information from the provider to be inspected. CQC also gathered routinely collected feedback from service users, including feedback collected through local surveys and, in the case of the acute and specialist mental health sectors, nationally collated feedback.^29^, ^30^

From our interviews, we heard how an effort to publicize their ratings and reports in the media had resulted in an increased awareness among service users and the wider public of CQC's work. Changing attitudes to reporting poor care were leading to an increase in individuals contacting CQC directly with concerns and complaints.

*…when we first started doing GP inspections we had hardly any ‘share your experience’ information from the public, hardly any whistleblowing. What's been interesting as we've gone through, and I don't know whether that's because we've done the press releases, because we've proactively used that, because we've publicised when we've done reports, I don't know, but we've started to see that the volume has increased significantly*. *(CQC staff, general practice, area E)*

In addition to individuals reporting experiences to CQC, we found that representatives of service user groups and voluntary sector organizations were routinely invited to report any individual or collective experiences they had collected during their own activities to engage service users and the public. We heard that such organizations were contacted via email or by telephone by a CQC inspector in advance of inspection. However, we found that the effectiveness of this varied by organization and was dependent on the amount and quality of the information held and the capability and capacity of the groups to interpret it and respond to CQC.

*[CQC] contacted us asking us to share experiences with them. We have had quite a lot of data anyway, both in county residents and city residents. And so what we did is rather than, sort of, say, here's our feedback we went through each experience and we looked at the core services that the CQC inspect against and we applied that to the data that we had so it would be useful for them and so they could say, well, okay, let's look at – I don't know – urgent care and see what the feedback says. And we produced a report*. *(Healthwatch, area E)*

*I just do a copy and paste really from the previous one saying, we don't have any information about that. Good luck with your inspection. So they do talk to us quite a lot, it's just that I don't have anything to come back to them with nine times out of ten, you know*. *(Healthwatch, area F)* .

We found that these data were used to inform the subsequent inspection. The information was compiled into a data pack to be used by the inspection team to inform their planning and focus their evidence gathering.

*If you're in a hospital, whether it'll be mental health or acute, you have reams and reams and reams of intelligence data, whether that's patient surveys, whether that's…well, you know the wealth of information that hospitals can produce. For adult social care it's so, so limited but we are very reliant on what we can gather from a local authority and their commissioners and CCGs and from people who use services or their carers*. *(CQC staff, adult social care, area F)*

Our review of CQC documents found that the information obtained through these activities also contributed to CQC's “intelligent monitoring” process, helping CQC to determine what to inspect, when, and where to focus their attention.^29^, ^30^

### 3.2 Consulting service users: seeking individual voice

In addition to gathering available information about service users’ experiences from individuals and representatives of service user groups, we found from our document review and interviews with CQC staff that some specific engagement activities were organized around the time of inspection. These engagement activities were designed to elicit information *about individuals’ experience of care,* tailored to CQC's “key lines of enquiry.” CQC inspectors would speak with service users and their carers in listening events organized in the local community. In the acute and mental health sectors, feedback was also sought through focus groups, drop in sessions and home visits.^29^, ^30^ The information generated from these activities was used to inform the ongoing inspection.

Our interviews with representatives of service user and citizen groups highlighted some practical issues with these engagement activities. We were told that service users and citizens wanted to contribute but the events were often not well publicized and organized without enough advance notice for certain groups to attend, particularly those who might require assistance. Generally, there was a perception from interviewees that opportunities to engage were not sufficiently considerate of the intended participants and potentially demonstrated a lack of knowledge or understanding of the local context.

*…to get a group of people with learning disabilities to engage about a topic, you actually need probably six to eight weeks. Even with the group that you've got regularly running who are quite au fait with lots and quite vocal, you still need some lead‐in time…* *(Voluntary organisation, mental health, area A)*

*From here, it's quite a complicated journey and it's not somewhere where we'd normally be going at all. It was my view that they should have looked at the general spread of patients going to [the hospital] and had maybe as many as three [events]*. *(Voluntary organisation, area A)*

We heard differing perceptions of the listening events from interviewees. On the one hand, we were told they sometimes attracted those with a particular, often negative, experience to share, but we also heard that while that may be the case for some individuals, on the whole there were a range of experiences voiced at these events.

*Engaging with the public is really different because it's quite difficult for them to engage on a positive front. So if we hold a listening event it doesn't mobilise the people largely who had a good or an okay experience of the trust. It will very often mobilise those people who have [negative experiences]* *(CQC staff, acute, area B)* .

*Obviously at these meetings, you know, people have a range of issues, but my experience is that, whilst you get an odd patient who has a very personal axe to grind, generally people put very sensible points and you have a worthwhile exchange. But, how that translates into the inspection process, remains rather mysterious as far as I'm concerned*. *(Voluntary organization 2, area A)*

### 3.3 Involving service users in inspection: experts by experience

Service users had some citizen power when they participated in CQC inspections as “experts by experience.” These service users were recruited at a national level on behalf of CQC by two large contracted organizations, Remploy and Choice Support. They took part in inspections as full members of inspection teams and spoke with service users and their carers during the inspection to hear the user voice. There were mixed perceptions among our interviewees about the use of experts by experience as a way to elicit patient experiences.

We heard from a service user representative perspective that involving experts by experience was important, as those using services brought greater insight, and that involving experts by experience helped to incorporate multiple perspectives on care. However, there was a perception among some of our interviewees sampled from patient and public representative groups, who had experience of undertaking inspections, that the individual lived experiences of some experts by experience could colour or affect their contributions.

*I think it mainly helps the inspector, you know. And also …they might have a better idea of the questions they need to ask, in order to assess whether somebody is satisfied and is having their needs met. But I suppose there's a danger there that they might bring too much of their own experience into things. If an expert by experience has had a bad experience, I suppose that could colour the sorts of questions and the way they ask those questions, couldn't it?* *(Voluntary sector organization, area F)*

We heard that the background and experience of experts by experience was often not very relevant to the services or providers they were involved in inspecting. It was not clear from this data whether the involvement of experts by experience was serving the intended purpose.

*…experts by experience are far better at challenging professionals and holding up a mirror of reality to professionals than going out and talking to other people who use services. I think there's a bit of a dynamic there that often people who use services don't want to talk to somebody else who use services. They want to talk to a proper inspector*. *(Service user group, CCG, area C)*

It emerged from our data that many service user group and voluntary sector representatives were unaware of the use of experts by experience in inspection. One interviewee from a local Healthwatch spoke of how well positioned they were to support this role, but they were not able to be involved in providing experts by experience for inspections in their area.

*Experts by Experience service they provide is so aligned with what we do. …We're part of a collaborative, a fairly large charity which is well positioned to take on quite a big bid, but not to the scale they were talking about. I think there's four or five contracts nationally they've awarded, so we just won't be able to do it. We spent a lot of time building a collaboration around that which then didn't come to anything*. *(Healthwatch, area B)*

### 3.4 Speaking with local service user groups: seeking collective voice

In addition to gathering individual experiences, and facilitating engagement regarding a specific, forthcoming inspection, CQC engaged with voluntary organizations, representatives of service user groups and other stakeholder groups. Such attempts to gather a collective patient voice were at the higher levels of the participation ladder. Examples included CQC's “Tell us about your care” partnerships with a number of major national charities (such as Carers UK, Mind and the Patients Association), as well as discussions and interactions with local Healthwatch, local overview and scrutiny committees, NHS and other complaints advocacy services, and identified patient representatives at Clinical Commissioning Groups and health and wellbeing boards.

*In the run up to the [hospital name] inspection again we would have got a list of the key organisations within each of the boroughs and we would have attended meetings and met with groups of service users who would again also have the opportunity to tell us about their perspective of the services*. *(CQC staff, mental health, area A)* .

We also found that CQC sought advice through some standing advisory panels (such as “eQuality Voices” for diversity and quality, Service User Reference Panel (SURP) for those detained under the Mental Health Act, “SpeakOut” for diverse and vulnerable communities).^30^ These groups and networks served as a way to bring voices of smaller groups, into much larger networks. These networks raised awareness of and developed relationships between CQC and local groups and organizations across the country. The collective voice was then used by CQC to prioritize forthcoming inspections and informed the areas of focus for inspection teams.

*…actually we're also involved in the Speak Out network, for smaller organisations, with the CQC. We've been involved in that ever since it was set up …as well as us organising a kind of focus group for people locally, to talk to the CQC prior to them going into [hospital name] and inspecting*. *(Voluntary organisation, mental health, area A)*

We found there were variations in how this was working between local areas. In areas where relationships were more established between CQC and service user groups, there were more avenues for user voices to be heard, and fed back. Variation in capacity and capability to facilitate opportunities for people to engage with CQC was also reported between groups and organizations.

*One of the things we've developed in some patches, and that's more because the managers and the teams have been around longer, is engagement at a local level with local groups, so local community groups*. *(CQC staff, adult social care, area F)*

*…it's an issue and I'm, kind of, aware that we probably could do more work with the CQC to be honest if we had more resources, but there's not many of us and we've been focused on other things*. *(Healthwatch, area E)*

We heard that groups and organizations were exposed to a range of service user experiences in their work.

*We do outreach sessions to specific groups of people who want us to come along and tell us their views. …So we go along there regularly and we sit down and we say, so what's going on? Have you had any bad experience? Good experiences as well, you know. Anything coming to light that you think I need to investigate. …We don't hold meetings here and expect people to turn up*. *(Healthwatch, area F)*

*So on a daily basis we come in contact with an awful lot of people who are involved in care services and often they will talk to us about their experience of using those services*. *(Voluntary organization, adult social care, area F)*

Yet when asked, many, including the two interviewees above, said the group or organization they represented did not have an established relationship with CQC.

### 3.5 Feedback and follow‐up after inspection

Our analysis found that many of those who contributed their voice to engagement activities, either, through a service user group or voluntary organization or by participating in the inspection, did not subsequently receive feedback from CQC about the outcomes of the inspection.

*You don't see evidence that oh, we had four bits of feedback about this, however, as a result of that feedback we went and we did a spot inspection. You don't see that model followed I don't think*. *(Voluntary organization, adult social care, area A)*

*I would have thought that, at the very least, the CQC, having invited people to meetings to help them conduct the inspection, you know, should take their details and then get back to them when the inspection report has been done, and say, look these were our findings, or even headlines and you can see the rest on our website, or something. I know they don't do that at all, in my experience*. *(Voluntary organization 2, area A)*

Multiple interviewees also told us they wanted to hear how their voice had been translated into the inspection process and how their input had resulted in change or action, rather than this being a “mysterious” process, as one interviewee put it.

*Yeah, and I think people want to hear, actually how their views might have been acted on, or, ‘cause that's the thing isn't it, that if people give their views, then actually they want feedback about that, they want that written up, they want to hear how that will change something, and then they want to see that change*. *(Voluntary organization, mental health, area A)*

We also found that sometimes people disagreed with the inspection findings or outcome when they did hear about it and wanted to voice this but did not know how to.

*Certainly in the patient group that I'm in with my GP, when the outcome of the CQC report came out, the patient group were not in agreement and were really disappointed and wanted to voice their support of the surgery and say, well this is what we think. …I think one of the issues was how could we voice our opinions and say, you know, we don't necessarily agree with that, or that's not been our experience*. *(Service user group, CCG, area F)*

*It was only post‐event that then people wanted… and then post‐publication that people then wanted to contribute. And at that stage then it's not particularly useful to us and, you know, the motivations for why that is I don't know, you know, so perhaps they either agreed or didn't agree with our findings*. *(CQC staff, mental health, area D)*

Care Quality Commission engagement activities were mainly designed to enable and gather voice prior to the inspection rather than to support engagement afterwards. We found from our interviews and learned through our observations that for the acute and mental health NHS trusts, CQC held a quality summit to present its inspection report and the provider's response and action plan. Many stakeholders were invited to attend, but at this stage there was not really any scope to influence the inspection findings or outcomes.

*So all the groups of people that didn't agree with the outcomes of the report ….I'm not robbing them of that, that's their experience but what the quality summit did achieve was allow us to do some PR about our processes and systems*. *(CQC staff, mental health, area D)*

Some suggestions for routes to feedback were provided by interviewees who said this could be done at events, in a newsletter, or by having their comments visible in the report. Ongoing engagement between CQC and patients/services users and the public would foster a two‐way sharing of information and greater enable voice.

*If there was a short newsletter attached to that that could go out to the clients that would be really great, because what you tend to do sometimes is give your information, but the loop doesn't close, so you sit and you give the information, but you don't actually get the feedback*. *(Service user group chair, acute, area E)*

## 4 DISCUSSION

Care Quality Commission has a large remit, tasked with regulating all health and social care providers in England, but limited resources. To do this, it relies on information held by many stakeholders and system partners, including patients, service users and the public. The overhaul of CQC's regulatory regime in 2013 brought with it opportunity for service user voice to have a greater role in regulatory activities. From a review of key policy documents, interviews with CQC staff and patient and public representatives, we have found that CQC conducted various activities to include the service user voice within inspection and rating. Since the completion of our fieldwork in 2017, CQC has continued to develop its approach to user engagement.^31^

Our findings highlight how difficult it can be to involve service users in health and social care regulation. National regulators are typically large, bureaucratic organizations with a culture that emphasizes consistent authoritative application of rules by inspectors, who should maintain some distance in order to be objective and avoid capture. While there are potential benefits, being responsive to local communities and their concerns makes the regulator‐regulatee relationship more complex, placing additional demands on regulatory staff, who need to adopt a more flexible approach that is socially and politically aware, in order to engage service users in a productive process.^32^ Our study also adds to the literature which has highlighted ongoing difficulties in involvement of health and social care users in England, particularly at a collective level.^33^ CQC has shown that progress can be made, but that institutionalising and sustaining change may be difficult. There may be a need to go beyond the middle levels of the ladder to work in partnership with service user groups in order to enable fundamental and lasting change.

Despite the strengths of our study, it was not without its limitations. We conducted 61 interviews which enabled us to comment on the role of service user voice within the inspections. We interviewed a range of CQC and service user and voluntary organization representatives across six case study areas and four health and social care sectors. Systematic variations between our case study sites were not a prominent feature of our data analysis and so are not specifically reported on in our findings, future research may seek to explore variations further. Our study did not explore CQC's public engagement work at a national level to support its thematic reviews such as the State of Care reports.^34^

Interviewing the public, service users and experts by experience directly could provide more detailed understanding of how these engagement activities work in practice. Further study could focus on the role of experts by experience, to understand how local recruitment might work in practice.

## 5 CONCLUSIONS

The encounters between CQC, individual and collective voices seemed to be somewhat transactional, organized directly to serve CQC functions and processes but not to build enduring relationships with local service user groups. There was a lack of transparency about how voice was incorporated into the inspection and rating process, and once people had shared their experiences with CQC, the engagement came to an end. Developing relationships that exist beyond an inspection and outside the inspection cycle would create opportunities for mutual and ongoing sharing of information, which could be used to help assess risk and build detailed profiles of providers so at the point of inspection, teams have more service user data to draw upon, and are better placed to engage in more focused and appropriate service user involvement during inspections.

## CONFLICT OF INTERESTS

There are no conflict of interests.
